# Effects of plastic mulching on soil CO_2_ efflux in a cotton field in northwestern China

**DOI:** 10.1038/s41598-022-08793-x

**Published:** 2022-03-23

**Authors:** Zhimin Zhao, Fengxia Shi, Fachun Guan

**Affiliations:** 1 School of Politics and History, Ningxia Normal University, Guyuan, 756000 Ningxia China; 2 Assets Department of Ningxia, Normal University, Guyuan, 756000 Ningxia China; 3 Jilin Academy of Agricultural Sciences, Changchun, 130033 Jilin China

**Keywords:** Ecology, Climate sciences, Ecology, Environmental sciences

## Abstract

In Northwestern China, more and more traditional cultivation system (TC) with no mulching and flood irrigation have been replaced by modern cultivation technology (MC) combining plastic film mulching with drip irrigation. Does plastic film mulching increase or reduce soil CO_2_ emission in arid areas? In order to study the effects of plastic mulching on soil CO_2_ efflux, a field study was conducted to compare soil CO_2_ concentration, soil CO_2_ efflux, soil temperature and moisture between the TC treatment and the MC treatment during a cotton growing season in Northwestern China. The seasonal patterns of soil profile temperature and soil moisture in the TC treatment were similar to that in the MC treatment. The mean value of soil profile temperature in the MC treatment was higher than that in the TC treatment. Except for soil moisture at 15 cm depth, the mean value of soil moisture at 5 cm and 10 cm depths in the MC treatment was higher than that in the TC treatment. The variation patterns of soil CO_2_ concentration and soil CO_2_ efflux in MC treatment were different to that in the TC treatment. Although the peak of soil CO_2_ concentration in the TC treatment was earlier than that in the MC treatment, the duration of soil CO_2_ concentration with high values in TC treatment was shorter than that in the MC treatment. Based on the model of Fick’s first diffusion law, soil surface CO_2_ efflux in the MC and TC treatments were determined. The surface CO_2_ efflux in the TC treatment calculated by Fick’s first diffusion law model was in good agreement with the value measured by chamber method. The seasonal curve of soil surface CO_2_ efflux in the MC treatment indicate the similar pattern with that in the TC treatment, and the rate of CO_2_ efflux was lower in the MC system. In the MC treatment, the seasonal variation of soil surface efflux was explained more by soil moisture than by soil temperature. However, in the TC treatment, the seasonal variation of soil surface efflux was explained more by soil temperature than by soil moisture. Over the completely experimental period, accumulated rates of soil CO_2_ efflux were 361 g C m^−2^ and 474 g C m^−2^ for the MC and TC system, respectively. We concluded that converting agricultural practices from traditional cultivation to the plastic mulching cultivation could reduce soil CO_2_ efflux by approximately 110 g C m^−2^ year^−1^ in agricultural land in arid areas of Northwestern China.

## Introduction

Soils contain the largest pool of terrestrial organic carbon pool in terrestrial ecosystems^1^, storing 1500–2000 Gt of organic C^2^. Moreover, the global CO_2_ flux from soils ranges from 64 to 72 Gt Cy^−1^, which accounts for 20–38% of annual emission of CO_2_ from terrestrial and marine sources to the atmosphere^3–5^. Greenhouse gas (GHG) emissions contribution to global climate change and are influenced by land use and agricultural practices^2,6^. Improper agricultural practices and land use (e.g., deep flowing irrigation and nitrogen over-application) were estimated to contribute one third of anthropogenic GHG emissions^7–9^.

Traditional cultivation (TC) involving flood-irrigation with no mulching is the main cropping system used in dry-land agriculture in northwestern China^10^. However, shortages of irrigation water and low temperatures in spring have become critical factors limiting the productivity and sustainability of such cropping systems^11^. Modern cultivation technology (MC), combining plastic film mulching with drip irrigation, can improve soil temperature, irrigation water efficiency^12,13^ and crop yield.

MC planting is widely promoted in China^12,13^. In 2009, the land area of MC planting in Northwest China reached 1 million hectares^14^. However, mulching alters the soil microenvironment and has a significant impact on the carbon cycle processes^15^, resulting in changes in soil CO_2_ production and CO_2_ efflux^16,17^.

Understanding the impact of mulching on soil CO_2_ efflux is of great significance for formulating carbon management strategies in dry-land areas of China. However, since the plastic mulching changed soil temperature, soil moisture and the gas exchange between soil and atmosphere, the impact of mulching on soil CO_2_ efflux is complicated. Some studies^18–20^ have shown that the increasing of soil temperature and moisture caused by plastic film mulching enhanced soil CO_2_ production. Moreover, some studies^21–23^ showed that the pore-space in soil profile acts as a “buffer” for soil CO_2_ efflux. The plastic film mulching affected the diffusion of CO_2_ in the soil profile due to influencing soil profile CO_2_ concentration. Then, how does the plastic film mulching influence soil CO_2_ efflux in arid region? Considering the difficulty of measuring CO_2_ efflux from the soil surface without interfering with plastic film mulching and the effects of plastic film mulching on diffusion of CO_2_ in soil profile and soil environment, it is difficult to directly explore the impact of plastic mulching on soil CO_2_ efflux, such as chamber method. In order to solve this problem, we should not only explore the feedback of CO_2_ in the soil profile to the plastic film mulching, but also explore the effect of plastic film on soil environmental conditions. The variation of CO_2_ concentration in soil profile can indirectly reflect the variation of soil environmental conditions and the diffusion of CO_2_ in soil profile. Therefore, it is effective to study the effects of plastic mulching on soil CO_2_ efflux by measuring and analyzing the variation of CO_2_ concentration in soil profile. Soil temperature and moisture are general considered to be the two most important factors controlling soil CO_2_ production^24^. To analyze the influence of plastic film on soil environmental conditions, soil temperature and moisture are two fundamental factors to be considered. Based on the variations of soil profile CO_2_ concentration, soil temperature and moisture and soil physical properties, Fick’s first law of diffusion model can be used to simulate soil profile CO_2_ efflux. Some studies showed that soil CO_2_ effluxes measured by Fick’s first law of diffusion are well related to those by chamber method^25,26^. The aim of the study was to explore the effect of plastic film mulching on soil CO_2_ efflux in Northwest China and its causes through Fick’s first diffusion law model. The basic assumption of this study is that plastic film mulching will reduce soil CO_2_ efflux.

## Materials and methods

### Site description

**Figure 1:**
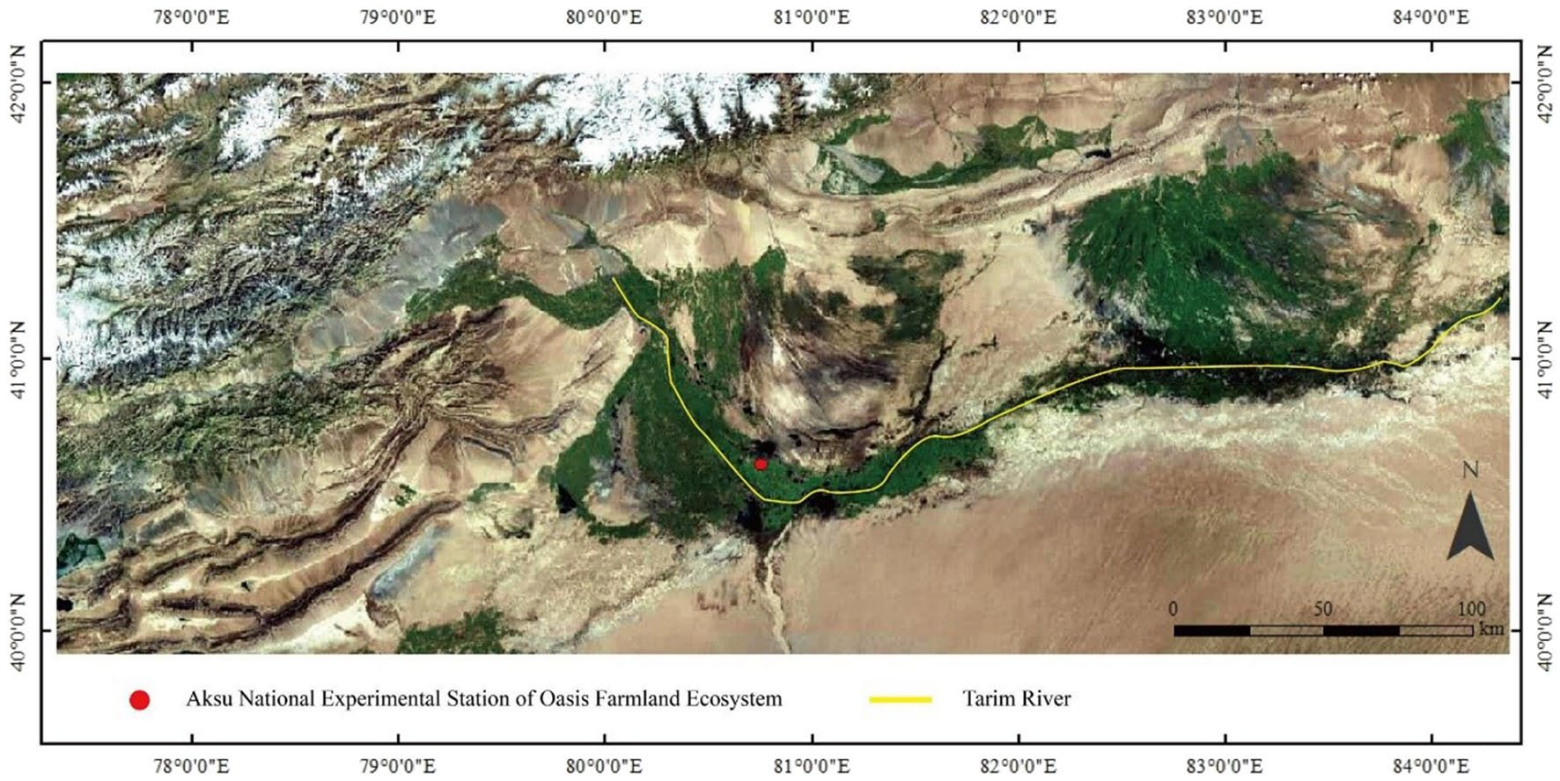
Location of the Aksu National Experimental Station of Oasis Farmland Ecosystem (the map was created by software: QGIS Version 3.16.15 LTR: URL, https://www.qgis.org/en/site/).

In 2012, a field experiment was conducted in the Aksu National Experimental Station of Oasis Farmland Ecosystem^27^ (40°37′ N, 80°45′ E, altitude 1028 m) (Fig. 1), located in the west of Tarim River Basin in Xinjiang Province, China. The experimental area had a typical temperate arid climate. During the study period (May to October), the average minimum and maximum temperatures varied between 16.7 and 34.8 ℃ respectively.

The cotton fields where the experiment conducted were public land, belong to Xinjiang Institute of Ecology and Geography, Chinese Academy of Sciences, China. With the permissions of Xinjiang Institute of Ecology and Geography, we conducted experiments in the cotton field of the Aksu National Experimental Station of Oasis Farmland Ecosystem.

### Experimental design

Two treatments, each 10 m × 10 m in size, were established on one of cotton fields at the Aksu National Experimental Station of Oasis Farmland Ecosystem on April 5, 2012.One treatment planting cotton with TC method, the other with MC method. For the MC method, a high-density and air-tight transparent polythene film (0.01–0.02 mm thick, 1.25 m wide) was placed over the soil surface before sowing. Small holes (0.02 m × 0.02 m, at 0.1 m intervals within a row) in the plastic film were made to place cotton seeds. Four rows were sown on each strip of plastic film. For the TC treatment, the plants were sown as that for the MC treatment. The planting density (266 667plant ha^−1^) and irrigation pattern (frequency and volume of irrigation) for the TC method were entirely consistent with those for the MC method.

Half-hourly measurements of soil CO_2_ efflux, soil temperature and moisture were made on 6 June 2012. The whole experiment was completed on 4 November 2012. According to irrigation, the whole experiment can be divided into three stages: stage before irrigation (from 6 to 24 June), during irrigation (from 25 June to 10 October) and irrigation stop stage (from 11October to 4 November). During the irrigation period, we conducted seven times of irrigation (once in two week). The water-soluble compound fertilizer (N + P_2_O_5_ + K_2_O ≥ 51%) was used for fertilization in the experimental field, and the application rate was 30 g m^−2^. We dissolved water-soluble compound fertilizer in water and sprayed into the field by sprayer. During the irrigation period, the fertilizer was applied for 5 times.

The cottonseeds we used in this study comply with the provisions of the regulations of the People's Republic of China on Seed Administration and the detailed rules for the implementation of crop seeds. The fertilization we used in this study comply with the provisions of the People's Republic of China on Chemical fertilizer standard. All the experiments we conducted in the cotton field of Aksu oasis farmland ecosystem National Experimental Station met the provisions of the agricultural law of the People's Republic of China. We also carried out the experiment of this study under the guidance of the provisions of the measures for the administration of national field scientific observation and research stations.

#### Field measurement of soil CO_2_ concentration

Solid-state CO_2_ sensors (GMM221 and GMM222, Vaisala, Finland) were installed in the midpoint of each treatment to measure soil CO_2_ concentration. A cable connected each soil probe with a transimitter body placed on the ground. The transimitter sent output signals from the probe to a data logger (CR1000, Campbell Scientific Inc., Logan, UT, USA) and to an optional LCC display on the transmitter.

In each treatment, four CO_2_ concentration sensors were buried at depths of 0 cm, 5 cm, 10 cm and 15 cm. Soil CO_2_ concentrations were recorded once in 30 min. The measurement of soil CO_2_ concentrations were conducted from 6 June 2012 to 4 November 2012.On 8 November, these sensors were excavated and recalibrated in the laboratory. We found no change in the slope or offset.

#### Environmental and soil CO_2_ efflux measurements

The soil water content and temperature at the same soil depth with solid-state CO_2_ sensors were measured on the cotton fields at the Aksu National Experimental Station of Oasis Farmland Ecosystem^27,28^, respectively. Soil volumetric water content and soil temperature were measured using soil moisture probes (pF-Meter, EcoTech GmbH, Bonn, Germany)^26^ and temperature probes (PT100,Heraeus Sensor Technology, Kleinostheim, Germany)^26^, respectively.

Bulk density was determined by core method^29^. Briefly, a cylindrical metal sampler (volume of 100cm^3^) was inserted into the soil and carefully removed to preserve the sample. The sample was oven-dried at 105 °C and weighed. The ratio between dry weight of the soil sample and the cylinder volume was applied to provide the bulk density.

Half-hourly soil CO_2_ efflux measurements were conducted using a closed dynamic chamber method^26^ (CIRAS-1 PP Systems, Hitchin, UK) on the TC treatment, beginning on 6 June 2012. A chamber, with a diameter of 9.96 cm and a volume of 1, 170 cm^3^ was inserted into the soil at depth of 3 cm. Soil CO_2_ concentrations were measured by infrared gas analyzer. The collecting of CO_2_ from each sampling point took 120 s to get reliable estimates of soil CO_2_ efflux.

#### Data analysis

In order to calculate CO_2_ efflux in soil, Fick’s first law of diffusion was used:

1 $$F_{i} = - D_{s} \frac{dc}{{dz}}$$

where *F*_*i*_ is the CO_2_ efflux at depth *z*_*i*_, *D*_*s*_ the CO_2_ diffusion coefficient in the soil, and *d*_*c*_ / *d*_*z*_ the vertical soil CO_2_ gradient. In this study, the vertical CO_2_ gradient (dC/dz) was approximately a constant at different depths of soil in our site for the field conditions experienced in the TC treatment during study period. However, a quadratic function of depth to concentrations fitted to soil CO_2_ concentration gradients in the MC treatment.

*D*_*s*_ can be estimated as

2 $$D_{s} = \xi D_{a}$$

where ξ is the gas tortuosity factor and Da is the CO_2_ diffusion coefficient in free air. The effect of temperature and pressure on *D*_*a*_ is given by

3 $$D_{a} = D_{a} 0\left( {\frac{T}{293.15}} \right)^{1.75} \left( {\frac{P}{101.3}} \right)$$

where *T* is the temperature (K), *P* the air pressure (kPa), *D*_*ao*_ a reference value of *D*_*a*_ at 20 °C (293.15 K) and 101.3 kPa, and is given as 14.7 mm^2^ s^–1^^30^ .

There are several empirical models in the literature for computing *ξ*^31^. We used the Millington–Quirk model^32^:

4 $$\xi = \frac{{\alpha^{10/3} }}{{\phi^{2} }}$$

where *a* is the volumetric air content (air-filled porosity), *Φ* is the porosity. Note,

5 $$\phi = \alpha + \theta = 1 - \frac{{\rho_{b} }}{{\rho_{m} }}$$

where *ρ*_*b*_ is the bulk density, and *ρ*_*m*_ is the particle density for the mineral soil.

Soil surface CO_2_ efflux was calculated using the CO_2_ gradient flux method based on CO_2_ concentrations within the soil profile^1^. Briefly, the flux of CO_2_ between any two layers in the soil profile was calculated using the Moldrup model^33^.

In order to determine soil CO_2_ storage, the equation for CO_2_ was performed.

6 $${S}_{C{O}_{2}}=\frac{\partial (aC)}{\partial t}$$

where C (ppm) is the concentration of CO_2_ within the soil pores, $a$ is the aerial porosity of the soil layer, D is the molecular diffusivity of CO_2_ with the soil, and S(µmol m^−3^ s^−1^)is the source strength in the soil layer at depth.

We determined temperature responses for soil CO_2_ efflux using the van’t Hoff equation^34^ (Eq. 7);

7 $$R = R0e^{BT}$$

where R is soil CO_2_ efflux, T is soil temperature (°C) at 10 cm depth, and R_0_ is the soil respiration rate at a reference temperature of 0 °C (µmol m^−3^ s^−1^).

The *Q*_*10*_ value for Eq. (8) was calculated according to definition as:

8 $$Q_{{{1}0}} = R_{{{\text{T}} + {1}0}} /R_{{\text{T}}} = {\text{ e}}^{{{1}0{\text{B}}}}$$

where R_T_ and R_T+10_ are R_r_ or R_d_ rates at temperature T and T + 10, respectively. The *Q*_10_ value is independent of temperature in Eq. (8).

## Results

### Seasonal variation of soil profile CO_2_ concentration, soil profile temperature, soil profile moisture in the TC and MC treatment

During the experimental, soil profile CO_2_ concentrations, and soil profile temperature and soil profile moisture at 5 cm, 10 cm and 15 cm depths were simultaneously measured in the MC treatment and in the TC treatment.

**Figure 2:**
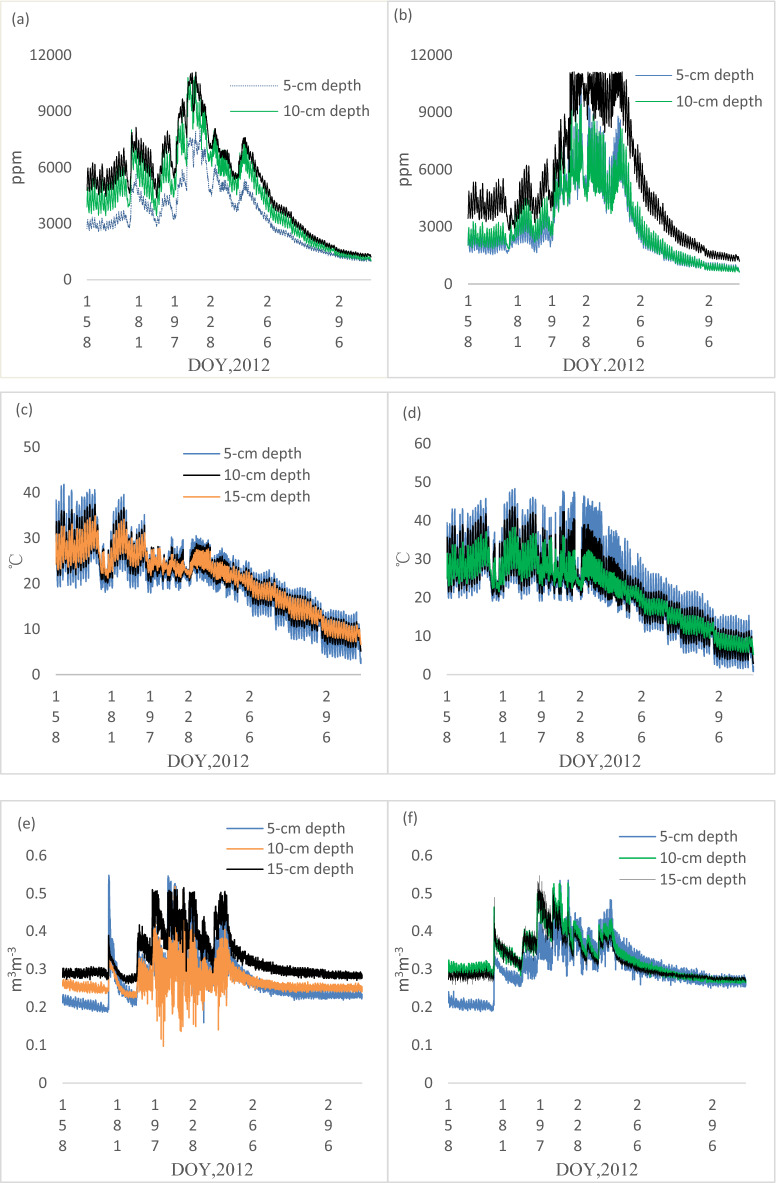
Seasonal variation of soil profile **(a)** CO_2_ concentration in the TC treatment, **(b)** CO_2_ concentration in the MC treatment, **(c)** temperature in the TC treatment, **(d)** temperature in the MC treatment, **(e)** moisture in the TC treatment, **(f)** moisture in the MC treatment.

Figure 2a,b presents CO_2_ concentration in the soil profiles during the experimental period. The seasonal pattern of CO_2_ concentration in the MC treatment was different from that in the TC treatment (Fig. 2a,b). Although soil profile CO_2_ concentration in the MC treatment fluctuated slightly, the general trend of soil profile CO_2_ concentration increased rapidly until end of Aug or early Sep, and then declined after early Sep. The general trend of soil profile CO_2_ concentration in the TC treatment increased rapidly until mid-Aug, and then declined after mid-Aug. The TC treatment had a peak in early Aug, which was earlier than the MC treatment (Fig. 2a,b). In general, the large differences in the soil CO_2_ concentration between the MC and TC treatments were observed from 25 Jun to 10 Oct, whereas only small differences were observed before25 Jun and after 10 Oct. In the TC treatment, soil CO_2_ concentration increased with soil depth reaching almost 11,092.52 ppm at 15 cm depth (Fig. 2a). Although soil CO_2_ concentration increased with soil depth in the MC treatment, the rate of soil CO_2_ concentration at 10 cm depth was slightly higher than that at 5 cm depth. Soil CO_2_ concentrations were higher at 15 cm depth in the MC treatment than in the TC treatment (Table 1). However, soil CO_2_ concentrations at 5 cm and 10 cm depths in the MC treatment were lower than that at 5 cm and 10 cm depths in the MC treatment, respectively (Table 1).

**Table 1:** The mean value of soil profile temperature, moisture concentration and soil surface CO2 efflux in the TC treatment and MC treatment.

Treatment	Temperature (℃)			Moisture (m^3^ m^−3^)			Concentration (PPM)			Efflux (μmol m^−2^ s^−1^)
	5 cm	10 cm	15 cm	5 cm	10 cm	15 cm	5 cm	10 cm	15 cm	0 cm
MC	22.63	22.01	21.72	0.303	0.331	0.323	2968	3068	5152	0.633
TC	20.42	20.79	21.03	0.274	0.271	0.333	3376	4451	5139	0.832

As Fig. 2c,d showing, the seasonal patterns of soil temperature and soil moisture in the TC treatment were similar to that in the MC treatment. The highest soil temperature was found in June. Overall, soil temperature was higher in the MC treatment than in the TC treatment, particularly during the irrigation stage (Fig. 2c,d).The mean value of soil temperature at 5 cm,10 cm and 15 cm depth in the MC treatment was higher than that in corresponding depth in the TC treatment, respectively (Table 1). However, the difference between the MC treatment and the TC treatment was not significant during the irrigation stop stage (10 October to 4 November). Although soil temperature at 5 cm, 10 cm and 15 cm depths fluctuated, the overall trend of soil temperature at 5 cm, 10 cm and 15 cm depths in the two treatments decreased (Fig. 2c,d).

Figure 2e,f showed soil moisture at 5 cm and 10 cm depths were greater during the experimental period in the MC treatment than in the TC treatment. However, soil moisture at 15 cm depth was slightly lower during the experimental period in the MC treatment than in the TC treatment (Fig. 2e,f).

### Seasonal variation of soil surface CO_2_ efflux in the TC and MC treatment

**Figure 3:**
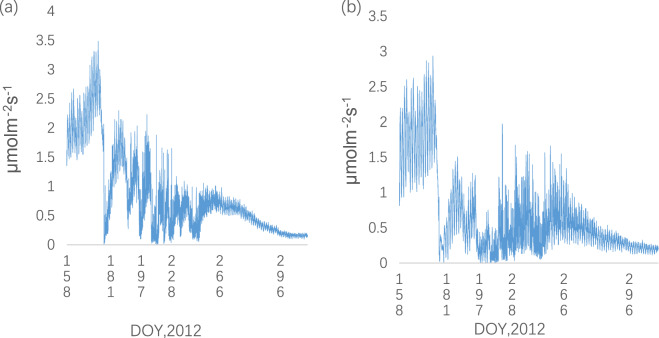
Seasonal variation of soil surface CO2 efflux **(a)** in the TC treatment **(b)** in the MC treatment.

Based on the equitation (6), the values of CO_2_ efflux at soil surface were calculated. There was a pronounced seasonal variation in soil surface CO_2_ efflux during the experimental period both in the TC treatment and in the MC treatment (Fig. 3). The seasonal curve of soil surface CO_2_ efflux in the MC treatment indicate the similar pattern with that in the TC treatment. The highest rate was found during the period before the irrigation stage from6 Jun to 24 Jun, and the lowest in end of Jul. Overall, the rate of soil surface CO_2_ efflux in the TC treatment was approximately 100% higher than in the MC treatment. The rate of soil surface CO_2_ efflux in the TC treatment ranged from 0.007μmol m^−2^ s^−1^ to 3.485μmol m^−2^ s^−1^ during the experimental period, whereas that in the MC treatment from 0.004μmol m^−2^ s^−1^ to 2.937μmolm^−2^ s^−1^. Total soil CO_2_ fluxes during the experimental period in the MC treatment and in the TC treatment were calculated, respectively. Total soil CO_2_ efflux during the whole experimental period was (30%) lower in the MC treatment (361 g C m^−2^) than in the TC treatment (474 g C m^−2^).

### Effects of soil temperature and moisture on soil surface CO_2_ efflux

**Figure 4:**
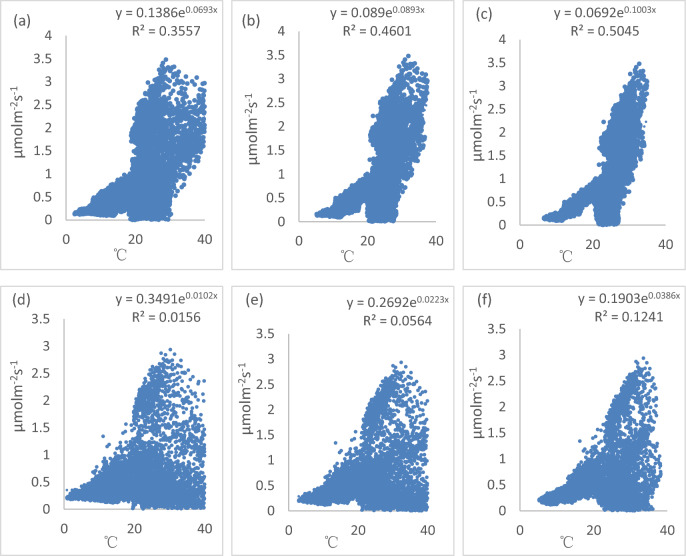
Correlations between soil surface CO_2_ efflux and soil profile temperature **(a)** at 5 cm depth in the TC treatment **(b)** at 10 cm depth in the TC treatment **(c)** at 15cm depth in the TC treatment **(d)** at 5 cm depth in the MC treatment **(e)** at 10cm depth in the MC treatment **(f)** at 15cm depth in the MC treatment.

By plotting soil CO_2_ efflux with soil profile temperature on one of cotton fields at the Aksu National Experimental Station of Oasis Farmland Ecosystem, the relationship between soil CO_2_ efflux and soil profile temperature was analyzed (Fig. 4). The exponential curves of soil CO_2_ efflux vs. soil temperature yielded *R*^*2*^ of 0.36 and *Q*_*10*_ of 2 at the depth of 5 cm, *R*^*2*^ of 0.46 and *Q*_*10*_ of 2.4 at the depth of 10 cm, and *R*^*2*^ of 0.5 and *Q*_*10*_ of 2.72 at the depth of 15 cm in the TC treatment. In the MC treatment, the exponential curves of soil CO_2_ efflux vs. soil temperature yielded *R*^*2*^ of 0.016 and *Q*_*10*_ of 1.107 at the depth of 5 cm, *R*^*2*^ of 0.056 and *Q*_*10*_ of 1.25 at the depth of 10 cm, and *R*^*2*^ of 0.12 and *Q*_*10*_ of 1.47 at the depth of 15 cm. The *Q*_*10*_ value increased with the depth of soil temperature measurements in both the TC treatment and the MC treatment. Higher *Q*_*10*_ was found when temperature was measured at the deep soil than that measured at the top soil in both the TC treatment and the MC treatment.

**Figure 5:**
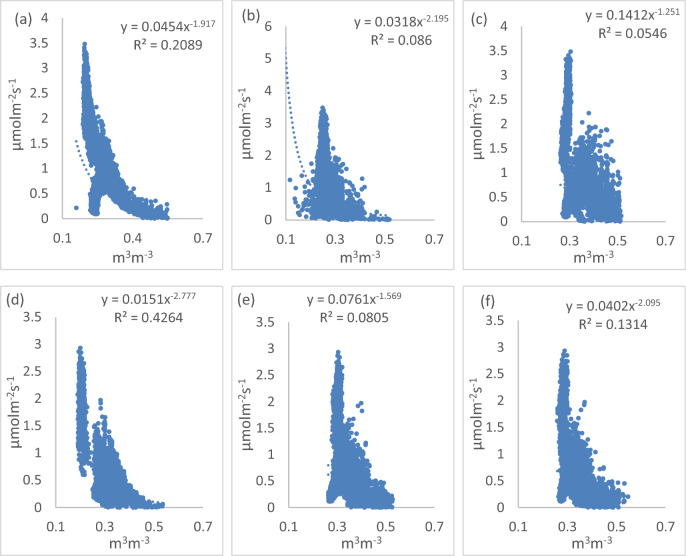
Correlations between soil surface CO_2_ efflux and soil moisture **(a)** at 5 cm depth in the TC treatment **(b)** at 10 cm depth in the TC treatment **(c)** at 15 cm depth in the TC treatment **(d)** at 5 cm depth in the MC treatment **(e)** at 10 cm depth in the MC treatment **(f)** at 15cm depth in the MC treatment.

By plotting soil CO_2_ efflux with soil moisture at different depths, we found that the correlation was the highest at the depth of 5 cm both in the TC treatment and in the MC treatment (Fig. 5). The power function curves of soil CO_2_ efflux vs. soil moisture yielded *R*^*2*^ of 0.2088 at the depth of 5 cm, *R*^*2*^ of 0.086 at the depth of 10 cm, and *R*^*2*^ of 0.0546 at the depth of 15 cm in the TC treatment. In the MC treatment, the power function curves of soil CO_2_ efflux vs. soil moisture yielded *R*^*2*^ of 0.4264 at the depth of 5 cm, *R*^*2*^ of 0.085 at the depth of 10 cm, and *R*^*2*^ of 0.13 at the depth of 15 cm. The highest correlation at 5 cm indicated that the soil moisture at this depth was suitable to study the relationship between CO_2_ efflux and moisture in both treatments (Fig. 5). In the TC treatment, the seasonal variation of soil CO_2_ efflux was well correlated with variations of soil temperature at 15 cm depth. However, the seasonal variation of soil CO_2_ efflux was well correlated with variations of soil moisture at 5 cm depth.

Overall, in MC and TC treatment, the correlation between soil surface CO_2_ efflux and soil temperature at 15 cm depth was greater than that between soil surface CO_2_ efflux and soil temperature at 5 cm and 10 cm depth. The correlation between soil CO_2_ efflux and soil moisture at 5 cm depth was greater than that between soil surface CO_2_ efflux and soil moisture at 10 cm and 15 cm depth. Furthermore, the correlation between soil CO_2_ efflux and soil temperature of the TC treatment was greater than that of soil CO_2_ efflux and soil moisture, and the correlation between soil CO_2_ efflux and soil moisture of the MC treatment was greater than that of soil CO_2_ efflux and soil temperature.

### Validation of CO_2_ efflux

**Figure 6:**
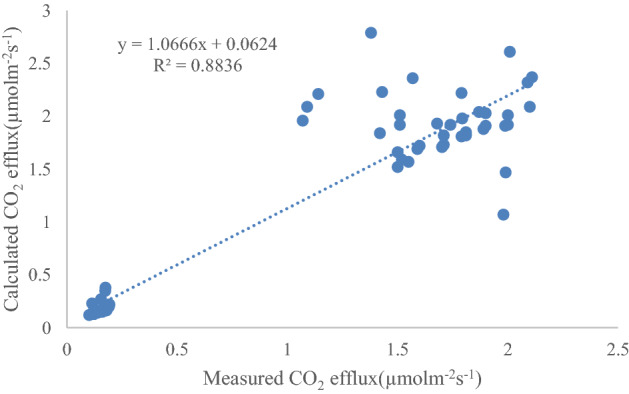
Comparison of calculated and measured CO_2_ efflux at soil surface (‘calculated CO_2_ efflux’ refer to CO_2_ efflux calculated by Fick’s first law of diffusion, and ‘measured CO_2_ efflux’ refer to CO_2_ efflux measured by chamber method).

To validate the estimated CO_2_ efflux results, we used simultaneous and manually measured data to compare with estimated ones (Fig. 6). However, CO_2_ efflux was only measured in the TC treatment during the period without irrigation. As Fig. 6 showing, a linear relationship was found between measured efflux and estimated with a slop = 1.0666, intercept = 0.0624, and R^2^ = 0.8836 .The estimated CO_2_ efflux is correlated well with measured data.

## Discussion

### Effects of plastic mulching on soil CO_2_ efflux

Proper agricultural practices and land use appear to minimize or reduce GHG emissions. Mulching reduced soil CO_2_ efflux compared with non-mulching in this study. This result was agreed by other studies^17,35^. The accumulated mean rate of soil CO_2_ efflux was 361 g C m^−2^ for the MC treatment and 474 g C m^−2^ for the TC treatment during the experimental period. The reduction in soil CO_2_ efflux result from mulching was 113 g C m^−2^, lower than the 152 g C m^−2^ reported by Okuda et al*.*^17^. Different soil types and vegetation at the two sites are partly responsible for the higher soil CO_2_ efflux in the study of Okuda et al.^17^. However, we inferred that the difference of experimental methods between the two studies was the main reason for the different results. Okuda et al.^17^ studied the effect of mulching by covered all the soil surface of the ridge, while in the present study we covered two thirds of the soil surface. Our result was similar with another study^35^. Li et al.^35^ concluded that the reduction in soil CO_2_ efflux result from mulching was 94 g C m^−2^, a slightly lower than that in the present study. The difference was probable due to the different climate and growth period of vegetation. Our experiment was conducted in the Aksu National Experimental Station of Oasis Farmland Ecosystem (80.75° E, 40.60° N), and the study of Li et al.^35^ performed in Fukang (87.45° E ,44.50° N). Because the climate at our study site was warmer than that in Fukang, the growth period of vegetation in our study site was longer than that in Fukang.

### Reasons of Mulching reducing CO_2_ emissions

**Figure 7:**
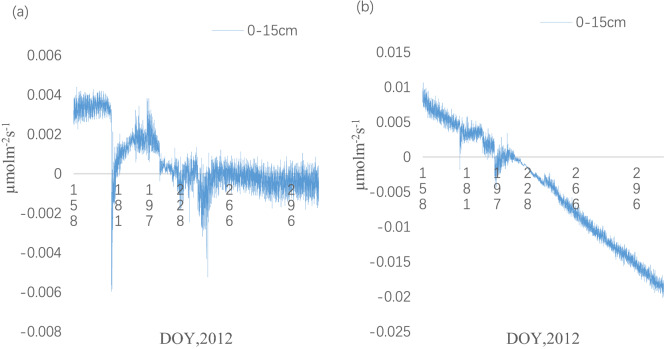
Seasonal variation of CO2 storage (0–15 cm) **(a)** in the TC treatment **(b)** in the MC treatment.

There are several reasons for the lower CO_2_ efflux in the MC treatment than in the TC treatment. One of the reasons is the barrier effects of the mulching on the gas exchange between soil and atmosphere. In this study, we found that CO_2_ concentration increased with soil depths in the TC treatment. However, CO_2_ concentration at 5 cm depth was similar with that at 10 cm depth in the MC treatment. Moreover, the value of CO_2_ concentration is mainly determined by the rate of CO_2_ production in a certain layer of the soil and by vertical diffusion of CO_2_ in and out of the layer if we neglect the horizontal transport^36^. Then we inferred that there were more CO_2_ stored in soil layer in the MC treatment than that in the TC treatment. In order to test this hypothesis, rates of change of CO_2_ storage in the 0–15 cm soil layer in both treatments were determined by Eq. (2). Although the time rate of change of CO_2_ storage was an order of magnitude smaller than the measured efflux in both treatments, the variation of CO_2_ storage (0–15 cm) in the MC treatment was larger than that in the TC treatment (Fig. 7).

Then, we concluded that the vertical soil CO_2_ gradient in the MC treatment was lower than that in the TC treatment. So, the result testified that the barrier effects of the mulching causing the lower soil CO_2_ efflux in the MC treatment. In addition, the cover of mulching film can decrease wind disturbance or turbulence, so as to reduce soil CO_2_ efflux. Some studies^8,37^ have shown that wind or turbulence can increase gas flux by increasing the diffusion rate of gas in soil.

Another important explanation for the lower CO_2_ efflux was that the mulching film changed soil environmental condition, such as soil moisture and temperature. Soil CO_2_ efflux results from the combination of CO_2_ production by autotrophic (root and mycorrhiza) and heterotrophic (decomposers) activities^38^, and then can transfer by diffusion through the porous medium in the soil. Keeping in mind that the effects of soil water content on production and transport of CO_2_ may influence the CO_2_ efflux in opposite directions, relating the efflux to soil water content is not simple^39,40^.

Some studies^17,28^ reported that the higher soil moisture would have decreased soil porosity and gas diffusivity, leading to the lower CO_2_ flux. The mean value of soil moisture at 5 cm depth in the MC treatment was higher than that in the TC treatment. Moreover, we also found that the correlation coefficient between soil CO_2_ efflux and soil moisture in the TC treatment was lower than that in the MC treatment (Fig. 5) . As a result, the decrease of soil CO_2_ efflux with the increase of soil moisture in the MC treatment was greater than that in the TC treatment. Furthermore, to a certain degree, higher soil moisture in the MC treatment would have decreased rewetting events, possibly resulting in a decreased CO_2_ efflux. Some studies have found that the rewetting of a dry soil can accelerate a large CO_2_ pulse^41–43^. Previous studies^44–48^ in open area have shown that soil CO_2_ flux is positively correlated with soil temperature, with increasing soil temperature accelerating soil CO_2_ flux, resulting in more CO_2_ emitted to the atmosphere. In present study, soil profile temperature (5 cm, 10 cm and 15 cm depth) in the MC treatment was higher than that in the TC treatment. However, the correlation between soil temperature and CO_2_ production may become weak in closed or partly-open areas such as under mulching conditions. In the present study, the correlation coefficient between soil CO_2_ efflux and soil temperature in the TC treatment was greater than that in the MC treatment (Fig. 4). As a result, the accelerated of soil CO_2_ efflux in the MC treatment was lower than that in the TC treatment. This result agreed by the other study^35^.

Based on the above analysis, the MC system has great potential to reduce CO_2_ efflux in Northwest China. However, many studies implicitly consider the measured soil CO_2_ efflux as the instantaneous soil respiration. In the short term, the CO_2_ efflux deviates from the soil respiration as soon as the amount of CO_2_ stored in the soil pore-space (SCO_2_/mol m^−2)^ is changing. The pore-space acts as a “buffer” for CO_2_. Then, it is possible that the reduction of soil CO_2_ efflux does not correspond to the reduction of soil respiration in the short term. In the long term, all CO_2_ produced in the soil must be emitted by the surface and soil CO_2_ efflux must correspond to soil respiration. In this study, the value of soil CO_2_ efflux in the MC treatment was higher than that in the TC treatment during the whole experimental period. We infer that the contribution of carbonate or silicate weathering to the lower CO_2_ efflux in the MC treatment cannot be neglected. This deduction agreed by the other study^48–50^.Then, exploring the effects of plastic mulching on soil CO_2_ efflux, it is also import to study the distinction between soil CO_2_ efflux and soil respiration. This issue needs to be study further.

## Conclusion

Modern cultivation technology (MC), combining plastic film mulching with drip irrigation reduced soil CO_2_ effluxes compared with traditional cultivation (TC). In this study, the accumulated mean rate of soil CO_2_ efflux was 361 g C m^−2^ for the MC treatment and 474 g C m^−2^ for the TC treatment during the experimental period.

Converting agricultural practices from traditional cultivation to the plastic mulching cultivation could reduce soil CO_2_ efflux by approximately 110 g C m^−2^ year^−1^ in arid agricultural land. The values of soil CO_2_ efflux in the treatment with plastic mulching cultivation were lower than that in the treatment with traditional cultivation.

Although plastic mulching cultivation reduces soil CO_2_ efflux than traditional cultivation, the possible reasons for this included the barrier effects of the mulching on soil CO_2_ efflux and changed soil environmental condition by the mulching film.

Consequently, although the result showed that plastic mulching cultivation reduces soil CO_2_ efflux than traditional cultivation in this study, the effect of plastic mulching cultivation on soil CO_2_ efflux changed with climate and method of plastic mulching.

In the future, we should study the effect of plastic mulching cultivation on soil CO_2_ efflux on the region with different climate and method of plastic mulching to enhance the precision of the estimated regional carbon.
